# Antimicrobial resistance and *Neisseria gonorrhoeae* multiantigen sequence typing (NG-MAST) genotypes in N. *gonorrhoeae* during 2012–2014 in Karachi, Pakistan

**DOI:** 10.1186/s12879-016-1673-1

**Published:** 2016-07-22

**Authors:** Kauser Jabeen, Pushpa Bhawan Mal, Erum Khan, Saeeda Chandio, Susanne Jacobsson, Magnus Unemo

**Affiliations:** Department of Pathology and Laboratory Medicine, Aga Khan University, Stadium Road, Karachi, Pakistan; WHO Collaborating Centre for Gonorrhoea and Other STIs, National Reference Laboratory for Pathogenic Neisseria, Department of Laboratory Medicine, Microbiology, Faculty of Medicine and Health, Örebro University, Örebro, Sweden

**Keywords:** *Neisseria gonorrhoeae*, Gonorrhoea, Antimicrobial resistance, Antimicrobial resistance surveillance, Extended-spectrum cephalosporins (ESCs), Ceftriaxone, Treatment, *N. gonorrhoeae* multiantigen sequence typing (NG-MAST), Pakistan

## Abstract

**Background:**

Globally, increasing antimicrobial resistance (AMR) in *Neisseria gonorrhoea* has led to decreased treatment options for gonorrhoea. Continuous monitoring of resistance is crucial to determine evolving resistance trends in *Neisseria gonorrhoea* and to suggest treatment recommendations. Quality assured gonococcal AMR data from Pakistan are mainly lacking. This study was performed to determine prevalence and trends of gonococcal AMR and molecular epidemiology of local strains during 2012–2014 in Karachi, Pakistan.

**Methods:**

Gonococcal isolates (*n =* 100) were obtained from urogenital specimens submitted to the Aga Khan University Laboratory, Karachi, Pakistan. Antimicrobial susceptibility was determined using Etest and molecular epidemiology was assessed by *N. gonorrhoeae* multiantigen sequence typing (NG-MAST). Quality control was performed using *N. gonorrhoeae* WHO reference strains C, F, G, K, L, M, N, O, and P, and ATCC 49226.

**Results:**

Susceptibility to spectinomycin, ceftriaxone and cefixime was 100 % and to azithromycin was 99 %. All isolates had low ceftriaxone MICs, i.e., ≤0.032 mg/L. Resistance to ciprofloxacin, tetracycline and penicillin G were 86 %, 51 % and 43 %, respectively. NG-MAST analysis identified 74 different sequence types (STs).

**Conclusions:**

A highly diversified gonococcal population, 74 NG-MAST STs (62 novel STs) with an increased resistance to penicillin G, ciprofloxacin and tetracycline circulated in Karachi, Pakistan. Fortunately, no resistance to ceftriaxone was detected. Accordingly, ceftriaxone can continuously be recommended as the treatment of choice. However it is recommended to increase the dose of ceftriaxone from 125 mg intramuscularly to 250 mg intramuscularly due to ceftriaxone MIC creep and emerging resistance reported in the region. Furthermore, due to the high level of resistance to ciprofloxacin (86 %) it is essential to exclude ciprofloxacin from the recommended first-line therapy. It is imperative to significantly broaden the gonococcal AMR monitoring with participation from other laboratories and cities in Pakistan.

**Electronic supplementary material:**

The online version of this article (doi:10.1186/s12879-016-1673-1) contains supplementary material, which is available to authorized users.

## Background

Global *Neisseria gonorrhoeae* incidence among adults is 78 million of the estimated 357 million cases of new sexually transmitted infections (STIs). Gonorrhoea is one of the most commonly reported STI with 111 cases per 100,000 population in the United States of America in 2014 [1–3]. Unfortunately, due to the lack of appropriate etiological diagnosis of gonorrhoea in many settings of Pakistan, no reliable figures regarding the incidence of gonococcal infections nationally exist.

*N. gonorrhoeae* was recently assigned as a “superbug” due to its ability to develop and retain *in vitro* and clinical resistance, i.e. resulting in treatment failures, to all antimicrobial agents available for treatment of gonorrhoea [2, 4–7]. The evolving resistance to ceftriaxone, the last treatment option for empirical therapy of gonorrhoeae has resulted in a fear of an era with extremely difficult to cure or possibly untreatable gonorrhoea [5–7]. Due to this emergent public health concern, the World Health Organization (WHO) in 2012 recommended a global action plan to halt the progression of *N. gonorrhoeae* antimicrobial resistance (AMR). This action plan strongly emphasizes the need of significantly enhanced surveillance of gonococcal AMR, using quality assured methods to monitor resistance trends, identify any emerging resistance and inform revisions of evidence-based treatment guidelines. The WHO has also suggested strong liaisons with other regional and national GASPs by re-launching the WHO Global Gonococcal Antimicrobial Surveillance Program (GASP) [7].

Despite these recommendations, surveillance of AMR in *N. gonorrhoeae* is difficult in settings where no national or regional GASP has been initiated. Inadequacy or complete absence of appropriate GASPs has been recently highlighted in Latin America, the Caribbean, Eastern Europe, Central Asia and Africa [2, 7–9]. This is certainly true also for the WHO Eastern Mediterranean Region (EMR). The WHO EMR includes the highly populated Pakistan with 182 million inhabitants, where no programme-based surveillance of gonococcal infections or AMR exists. In Pakistan, inadequate capacity to detect *N. gonorrhoeae* and perform quality assured AMR testing of *N. gonorrhoeae* in many laboratories are major factors that contribute to the absence of any national gonococcal AMR data. In small research studies, an increasing resistance to ciprofloxacin, penicillin and tetracycline in *N. gonorrhoeae* has been reported, but no resistance to extended-spectrum cephalosporins (ESCs) has yet been identified in Pakistan [10, 11]. However, in these studies very limited numbers of *N. gonorrhoeae* isolates were examined, and in general mainly qualitative disc diffusion methods have been used for AMR testing rather than the more reliable and quality assured quantitative methods, agar dilution and Etest, measuring the minimum inhibitory concentrations (MICs) of antimicrobials. In Pakistan, ceftriaxone 125 mg intramuscularly (IM) single dose (mostly used), cefixime 400 mg oral single dose or ciprofloxacin 500 mg oral single dose are currently the recommended first-line for empirical therapy of gonorrhoea. Alternative treatment option is azithromycin, 1 g oral single dose [12]. However, additional less effective antimicrobials such as penicillins and tetracyclines can also be used in both the private and public sector, and antimicrobials are freely available “over-the-counter” without prescriptions.

In addition to gonococcal AMR surveillance, understanding of the transmission of different gonococcal strains in the population is critical for effective control of gonorrhoea. *N. gonorrhoeae* multiantigen sequence typing (NG-MAST) [13] has been used globally to study prevalent strain genotypes, their transmission in sexual networks and core groups, and to describe distributions of AMR strains [14]. Although gonococcal prevalence surveys from Pakistan have reported rates ranging from 4.7 % to 20.2 % in high risk sex workers that suggest a high transmission in core groups, no molecular epidemiological data on high number of strains using NG-MAST is available [15]. A recent study evaluating AMR and genetic characteristics of gonococcal strains from Pakistan, India and Bhutan reported a highly diversified strain population [16]. However, this study included a low number (*n =* 18) of gonococcal isolates from Pakistan and recommended the need for expanded and ongoing surveillance with large number of gonococcal isolates in Pakistan.

The present study evaluated the prevalence and trends of *N. gonorrhoeae* AMR and described the genotypic distribution, using NG-MAST, of *N. gonorrhoeae* isolates cultured in 2012–2014 in Karachi, Pakistan.

## Methods

### Biological samples

The present research was conducted at the Aga Khan University (AKU) laboratories based in Karachi, Pakistan. The AKU laboratories comprise the largest laboratory network in Pakistan with satellite units in major cities and towns. The AKU Laboratory in Karachi was included as a sentinel site in the WHO GASP in 2012. Since then the laboratory regularly participates in the external quality assessment schemes of the WHO GASP with excellent result. In the current research, 100 mainly consecutive *N. gonorrhoeae* isolates cultured from urethral swabs (*n =* 85), high vaginal and cervical swabs (*n =* 14) and conjunctival swab (*n =* 1) as part of routine clinical care from January 2012 to February 2014 were included. *N. gonorrhoeae* isolates were identified by colony morphology, Gram staining, oxidase test, sugar utilization and Remel RapidNH® Panel (BioMerieux, France). These *N. gonorrhoeae* isolates have also been used in a study evaluating two different disc diffusion methods for susceptibility testing of *N. gonorrhoeae* (BMC Microbiology, accepted). All isolates were preserved in sterile 10 % glycerol phosphate broth at −70 °C and subsequently shipped with professional courier service (World Courier) on dry ice to Örebro University Hospital, Örebro, Sweden for the NG-MAST analysis. All cultures were performed according to standard protocol as part of the routine diagnostics and did not include patients’ identification. The study was approved by the Ethical Review Committee of Aga Khan University (Exemption #4052-Pat-ERC-16), and did not require informed consent as samples were taken as part of routine clinical care.

### Antimicrobial susceptibility testing

Antimicrobial susceptibility testing was done at the Microbiology laboratory of the Aga Khan University, Karachi, Pakistan. The MICs (mg/L) of ceftriaxone, cefixime, penicillin G, spectinomycin, ciprofloxacin, azithromycin and tetracycline were determined by the Etest (BioMerieux AB, Solna, Sweden) on GC agar base (Difco GC agar base medium, Becton, Dickinson, Sparks, MD, USA) with 1 % BBL IsoVitalex Enrichment (Becton, Dickinson, Sparks, MD, USA), according to the instructions from the manufacturer. MICs were interpreted using breakpoints for resistance, intermediate susceptibility and susceptibility according to the Clinical and Laboratory Standards Institute (CLSI) [17]. For azithromycin, breakpoints are not available as per CLSI, accordingly, the MIC breakpoints from the European Committee on Antimicrobial Susceptibility Testing (EUCAST; www.eucast.org) were applied. For quality controls, as recommended by the CLSI the *N. gonorrhoeae* reference strain ATCC 49226 was included in each testing. In addition, the *N. gonorrhoeae* WHO *N. gonorrhoeae* reference strains C, F, G, K, L, M, N, O, and P [18] were used as quality controls. β-lactamase production was identified by a chromogenic cephalosporin method using nitrocefin reagent (Oxoid, Hampshire, UK).

### Molecular epidemiological typing (NG-MAST)

Genomic DNA was isolated from cultured gonococcal isolates using the NorDiag Bullet robot (NorDiag ASA Company, Oslo, Norway) with BUGS’n BEADS STI-fast kit (NorDiag ASA Company, Oslo, Norway), according to the manufacturer’s instructions. NG-MAST [13] was performed as previously described [19]. NG-MAST allele numbers of the more variable segments of *porB* and *tbpB*, and sequence types (STs) were evaluated using the NG-MAST website (www.ng-mast.net). NG-MAST genogroups, comprising the main ST plus genetically associated STs, were assigned as reported in past studies. Briefly, a genogroup was defined as all STs which shared one allele and exhibited >99 % homogeneity in the other allele (≤5 bp difference for *porB* and ≤4 bp for *tbpB*) [20].

## Result

### Patient characteristics

*N. gonorrhoeae* isolates were grown from 86 (86 %) males and 14 (14 %) females. The mean age for the males was 30 years (median age: 29 years; range: 1.3 to 55 years) and for the females 29 years (median age: 29 years; range: 5 to 47 years).

### Antimicrobial susceptibility of *N. gonorrhoeae* isolates in 2012–2014 (*n* ***=*** 100) in Karachi, Pakistan

Summary of result of antimicrobial susceptibilities are given in Table 1.

**Table 1:** Antimicrobial susceptibility of *Neisseria gonorrhoeae* isolates (*n =* 100) in Karachi, Pakistan, 2012–2014

Antimicrobial	MIC range (mg/L)	MIC_50_ (mg/L)	MIC_90_ (mg/L)	S (%)	I (%)	R (%)
Ceftriaxone	0.002–0.032	0.008	0.016	100	0	0
Cefixime	<0.016	<0.016	<0.016	100	0	0
Spectinomycin	0.25–16	4	8	100	0	0
Azithromycin	0.016–4	0.094	0.25	99	-	1
Penicillin G	0.004–32	0.5	12	8	49	43
Tetracycline	0.12–96	3	24	12	37	51
Ciprofloxacin	0.25–32	4	14	0	14	86

*MIC* Minimum inhibitory concentration of an antibiotic necessary to inhibit the growth of a target organism, *MIC*_*90*_ Minimum inhibitory concentration of an antibiotic at which 90 % of the isolates were inhibited, *MIC*_*50*_ Minimum inhibitory concentration of an antibiotic at which 50 % of the isolates were inhibited, *S* Susceptibility; *I* Intermediate susceptibility, *R* Resistance

During 2012–2014, all (100 %) isolates were susceptible to ceftriaxone, cefixime and spectinomycin, and 99 % susceptible to azithromycin, that is, only one isolate was resistant to azithromycin (MIC = 4 mg/L). Furthermore, all isolates had MICs < 0.016 mg/L and MICs ≤0.032 mg/L of cefixime and ceftriaxone, respectively. However, the pattern of resistance the levels of resistance to ciprofloxacin, tetracycline and penicillin G were high that is, 86 %, 51 % and 43 %, respectively (Table 1). The resistance to ciprofloxacin (75–100 %), tetracycline (42–50 %) and penicillin G (25–42 %) during 2012–2014 remained high. Nevertheless, the resistance to penicillin G decreased from 42 % to 25 %, which was due to the decrease of β-lactamase producing isolates in 2014 (Table 2).

**Table 2:** Percentage (%) of antimicrobial resistance and β-lactamase production in *Neisseria gonorrhoeae* isolates (*n =* 100) obtained in Karachi, Pakistan, separated in 2012, 2013 and 2014

Antimicrobial	2012 (*n =* 48)	2013 (*n =* 40)	2014 (*n =* 12)
Ceftriaxone	0	0	0
Cefixime	0	0	0
Spectinomycin	0	0	0
Azithromycin	2	0	0
Penicillin G	42	42	25
Tetracycline	50	45	42
Ciprofloxacin	75	88	100
β-lactamase production	42	40	25

### *Neisseria gonorrhoeae* multiantigen sequence typing (NG-MAST)

In total, 94 (94 %) isolates could be successfully typed with NG-MAST. Remaining six (6 %) isolates were not revived due to contamination. Seventy-four different STs were identified and 62 STs (83.8 %) of these had not been previously described. The most prevalent STs were ST338 (*n =* 3 isolates), ST3328 (*n =* 3), ST10876 (*n =* 3), and ST10886 (*n =* 3). Twelve STs were represented by two isolates and 58 STs were represented by single isolates. In total, 36 *porB* alleles and 32 *tbpB* alleles were found. The most common *porB* alleles and *tbpB* alleles were *porB1173* (12 isolates), *porB90* (*n =* 11), *porB251* (*n =* 11), *tbpB136* (*n =* 12) and *tbpB121* (*n =* 10). ST338, ST3328 and ST10878 could be grouped in to the genogroup G338 (*n =* 8).

In Additional file 1: Table S1, the AMR of isolates assigned as the more prevalent NG-MAST STs is described. Out of the 74 identified STs, 33 (44 %) STs included *N. gonorrhoeae* isolates (*n =* 36) showing resistance to penicillin G, 43 (57 %) STs having resistance to tetracycline (*n =* 44), and 61 (81 %) STs displaying resistance to ciprofloxacin (*n =* 73). The only azithromycin resistant isolate (MIC = 4 mg/L) was assigned as ST8580.

## Discussion

This study showed that the susceptibility to ceftriaxone (100 %), cefixime (100 %), spectinomycin (100 %) and azithromycin (99 %) has remained high in Karachi, Pakistan. Accordingly, the results of the current study support using ceftriaxone as empiric treatment of choice in Pakistan for gonorrhoea, which has been recommended since year 2010 [12]. However, due to the increasing MICs of ceftriaxone and the emerging ceftriaxone resistance internationally the dose in Pakistan is recommended to be increased from 125 mg IM to at least 250 mg IM. Furthermore, because of increased resistance to ciprofloxacin (86 %) in Karachi it is important to exclude ciprofloxacin from the recommended first-line therapies. The reasons for the retained high susceptibility to ceftriaxone in Pakistan are unknown. This showed that gonococcal strain with decreased susceptibility to ESCs has neither been imported or if imported has not been successful to retain and establish local spread. However, it might also reflect that the ESCs resistance selection pressure has been low in gonococcal infections because ceftriaxone for therapy has only been recommended since 2010 (and instead ciprofloxacin was frequently used). In cases of ceftriaxone resistance, unavailability of ceftriaxone or severe β-lactam allergy, spectinomycin can be used as the second-line treatment. Azithromycin 2 g, should be added to the spectinomycin regimen in case of pharyngeal gonorrhoea, in accordance to European gonorrhea treatment guideline [21]. In current study, only one (1 %) azithromycin resistant isolate (ST8580) was found. Consequently, azithromycin remains effective against *N. gonorrhoeae* in Pakistan. However, due to the risk of selecting resistance (including high-level resistance) to azithromycin in *N. gonorrhoeae* [5]*,* as well as in etiological agents of other sexually transmitted infections, this antimicrobial should mainly not be used in gonorrhoea monotherapy and, if it has to be used (e.g., due to severe β-lactam allergy or resistance or unavailability of ceftriaxone and spectinomycin), azithromycin 2 g single oral dose should be used. Instead, it is recommended in the US [22] and European [21] gonorrhoea treatment guidelines that azithromycin should be added to ceftriaxone in the recommended first-line regimen. These dual antimicrobial therapies can be recommended in settings where ceftriaxone resistance has been verified or has not been excluded. Combined antimicrobial therapy will not only successfully eliminate gonorrhoea but also *Chlamydia trachomatis* infections and many *M. genitalium* infections. High levels of resistance to ciprofloxacin (86 %), tetracycline (51 %) and penicillin G (43 %) were identified in Karachi, Pakistan. Because of increased resistance to these antibiotics locally and also in most other countries worldwide, none of them ought to be suggested for empirical therapy of choice of gonorrhea in Pakistan or internationally [2, 5, 8–11]. Worryingly, in Pakistan penicillins, tetracyclines, fluoroquinolones, and macrolides are still commonly used as treatment options for gonorrhoea and these antibiotics are easily accessible “over-the-counter”. Due to this practice, it is crucial to substantially enhance the continuous monitoring of gonococcal AMR with a focus on emergence of resistance to ceftriaxone, spectinomycin and azithromycin, using MIC determination and also monitor treatment failures and gonococcal molecular epidemiological characteristics in Pakistan. It is therefore recommended to evaluate AMR annually with representation from more cities or regions to generate national data. It is also imperative to associate AMR to epidemiological data for development of effective control and prevention strategies. Consequently, financial assurance or support, both national and international, is crucial to emphasize continuous monitoring. The political commitment of the country leadership to curtail AMR in Pakistan and other countries in the WHO EMR will also be required.

NG-MAST has been used as a standard technique to evaluate clonal spread of gonococcal strains within high risk populations and to relate therapeutic failure with dissemination of particular strain types [14]. In the present study, 74 different NG-MAST STs among 94 isolates were identified. The high number of STs represented by only one isolate (*n =* 58) and STs that have not been previously described (62 novel STs) make the results hard to value. These results may be associated with the large catchment area, poor diagnostic capabilities in laboratories, lack sexual contacts tracing, and evolution of STs in Pakistan. Nevertheless, some NG-MAST ST clusters identified, e.g. G338 (*n =* 8), were associated with resistance to all three of the previously used antimicrobials ciprofloxacin, penicillin G and tetracycline. The successful ST1407 clone or genetically closely related STs belonging to genogroup 1407 associated with cefixime and ceftriaxone treatment failure internationally [5, 6], were not found in the present study.

The present study was performed on small number of strains which were collected as per physicians request and the researchers had no main control over patient selection. Therefore the results could not be generalized to the entire population. Additionally clinical data was not available from the patients to evaluate risk factors and treatment response. Despite these limitations, the results of this study are exceedingly important and will provide a baseline for future studies.

## Conclusions

A highly diversified gonococcal population, 74 NG-MAST STs (*n =* 62 novel STs) identified, with a high resistance to ciprofloxacin, penicillin G and tetracycline circulated in Karachi, Pakistan. Fortunately, no resistance to ceftriaxone was detected and ceftriaxone can continuously be recommended as the drug of choice. However it is recommended to increase the dose of ceftriaxone from 125 mg intramuscularly to 250 mg intramuscularly due to ceftriaxone MIC creep and emerging resistance reported in the region. Furthermore, due to the high resistance to ciprofloxacin (86 %) in Karachi it is essential to exclude ciprofloxacin from the recommended first-line therapies. It is important to emphasize the continuous monitoring of gonococcal AMR and investigate therapeutic failure in patients from other regions of Pakistan.

## Abbreviations

WHO, World Health Organization; AMR, antimicrobial resistance; GASP, gonococcal antimicrobial surveillance programme; WHO EMR, WHO Eastern Mediterranean Region; ESCs, extended-spectrum cephalosporins; MIC, minimum inhibitory concentration; NG-MAST, *N. gonorrhoeae* multiantigen sequence typing; AKU, Aga Khan University; CLSI, Clinical and Laboratory Standards Institute; EUCAST, European Committee on Antimicrobial Susceptibility Testing

## Additional file

Additional file 1: Table S1.
*Neisseria gonorrhoeae* multiantigen sequence typing (NG-MAST) STs and their resistance to antimicrobials in Karachi, Pakistan, 2012-2014. (DOC 45 kb)

## References

[1] Newman L, Rowley J, Vander Hoorn S, Wijesooriya NS, Unemo M, Low N (2015). Global estimates of the prevalence and incidence of four curable sexually transmitted infections in 2012 based on systematic review and global reporting. PLoS One.

[2] World Health Organization (WHO) (2012). Department of Reproductive Health and Research: Global action plan to control the spread and impact of antimicrobial resistance in *Neisseria gonorrhoeae*.

[3] Centers for Disease Control and Prevention. Sexually Transmitted Disease Surveillance, 2014. Gonorrhea statistics. Available at: http://www.cdc.gov/std/gonorrhea/stats.htm

[4] Chen SC, Yin YP, Dai XQ, Unemo M, Chen XS (2016). First nationwide study regarding ceftriaxone resistance and molecular epidemiology of *Neisseria gonorrhoeae* in China. J Antimicrob Chemother.

[5] Unemo M, Shafer WM (2014). Antimicrobial resistance in *Neisseria gonorrhoeae* in the 21st Century: past, evolution, and future. Clin Microbiol Rev.

[6] Unemo M, Nicholas RA (2012). Emergence of multidrug-resistant, extensively drug-resistant and untreatable gonorrhea. Future Microbiol.

[7] Ndowa F, Lusti-Narasimhan M, Unemo M (2012). The serious threat of multidrug-resistant and untreatable gonorrhoea: the pressing need for global action to control the spread of antimicrobial resistance, and mitigate the impact on sexual and reproductive health. Sex Transm Infect.

[8] Unemo M, Ison CA, Cole M, Spiteri G, Van de Laar M, Khotenashvili L (2013). Gonorrhoea and gonococcal antimicrobial resistance surveillance networks in the WHO European Region, including the independent countries of the former Soviet Union. Sex Transm Infect.

[9] Ndowa FJ, Francis JM, Machiha A, Faye-Kette H, Fonkoua MC (2013). Gonococcal antimicrobial resistance: perspectives from the African region. Sex Transm Infect.

[10] Zafar A, Jabeen K (2007). Antimicrobial resistance in *Neisseria gonorrhoeae* and limited treatment options. J Pak Med Assoc.

[11] Nizamuddin S, Jabeen K, Zafar A. Evaluation of predominant *Neisseria gonorrhoeae* strain types and its correlation with fluoroquinolone resistance in Pakistan. J Pak Med Assoc. 2011;61(5):446-9. 22204176

[12] Medical Microbiology & Infectious Disease society of Pakistan. http://www.mmidsp.com/wp-content/uploads/2012/06/Guidelines-for-Antimicrobial-Use-2.pdf

[13] Martin IM, Ison CA, Aanensen DM, Fenton KA, Spratt BG (2004). Rapid sequence-based identification of gonococcal transmission clusters in a large metropolitan area. J Infect Dis.

[14] Unemo M, Dillon JA (2011). Review and international recommendation of methods for typing *Neisseria gonorrhoeae* isolates and their implications for improved knowledge of gonococcal epidemiology, treatment, and biology. Clin Microbiol Rev.

[15] Sexually Transmitted Infections and HIV Among People With High Risk Behaviours: Results of Behavioural and Biological Surveys in Rawalpindi and Abbottabad, Pakistan. London School of Hygiene & Tropical Medicine. 2008.

[16] Sethi S, Golparian D, Bala M, Dorji D, Ibrahim M, Jabeen K (2013). Antimicrobial susceptibility and genetic characteristics of *Neisseria gonorrhoeae* isolates from India, Pakistan and Bhutan in 2007–2011. BMC Infect Dis.

[17] Clinical and Laboratory Standards Institute (2014). Performance standards for antimicrobial susceptibility testing; 24th informational supplement. CLSI document M100-S24.

[18] Unemo M, Fasth O, Fredlund H, Limnios A, Tapsall J (2009). Phenotypic and genetic characterization of the 2008 WHO *Neisseria gonorrhoeae* reference strain panel intended for global quality assurance and quality control of gonococcal antimicrobial resistance surveillance for public health purposes. J Antimicrob Chemother.

[19] Unemo M, Sjöstrand A, Akhras M, Gharizadeh B, Lindbäck E, Pourmand N (2007). Molecular characterization of *Neisseria gonorrhoeae* identifies transmission and resistance of one ciprofloxacin-resistant strain. APMIS.

[20] Chisholm SA, Unemo M, Quaye N, Johansson E, Cole MJ, Ison CA, Van de Laar MJ (2013). Molecular epidemiological typing within the European Gonococcal Antimicrobial Resistance Surveillance Programme reveals predominance of a multidrug-resistant clone. Euro Surveill.

[21] Bignell C, Unemo M, on behalf of the European STI Guidelines Editorial Board (2013). European guideline on the diagnosis and treatment of gonorrhoea in adults. Int J STD AIDS.

[22] Workowski KA, Bolan GA (2015). Sexually transmitted diseases treatment guidelines, 2015. MMWR Recomm Rep.

