# Supplementary material for: Antimicrobial resistance and Neisseria gonorrhoeae multiantigen sequence typing (NG-MAST) genotypes in N. gonorrhoeae during 2012–2014 in Karachi, Pakistan
Source: BMC Infect Dis. 2016 Jul 22;16:353. doi: 10.1186/s12879-016-1673-1 (PMC4957325; doi:10.1186/s12879-016-1673-1)
Supplement: Additional file 1: Table S1. — Neisseria gonorrhoeae multiantigen sequence typing (NG-MAST) STs and their resistance to antimicrobials in Karachi, Pakistan, 2012-2014. (DOC 45 kb) [file 12879_2016_1673_MOESM1_ESM.doc]

| NG-MAST ST | No. of isolates | Ceftriaxone  No. (%) | Cefixime  No. (%) | Spectinomycin  No. (%) | Penicillin G  No. (%) | Tetracycline  No. (%) | Ciprofloxacin  No. (%) | Azithromycin  No. (%) | β- lactamase No. (%) |
| --- | --- | --- | --- | --- | --- | --- | --- | --- | --- |
| 338 | 3 | 0 | 0 | 0 | 3 (100) | 3 (100) | 3 (100) | 0 | 3 (100) |
| 3234 | 2 | 0 | 0 | 0 | 0 | 1 (50) | 2 (100) | 0 | 0 |
| 3301 | 2 | 0 | 0 | 0 | 0 | 0 | 1 (50) | 0 | 0 |
| 3328 | 3 | 0 | 0 | 0 | 3 (100) | 3 (100) | 2 (67) | 0 | 3 (100) |
| 6079 | 2 | 0 | 0 | 0 | 1 (50) | 1 (50) | 2 (100) | 0 | 1 (50) |
| 6211 | 2 | 0 | 0 | 0 | 1 (50) | 2 (100) | 2 (100) | 0 | 1 (50) |
| 6323 | 2 | 0 | 0 | 0 | 0 | 0 | 2 (100) | 0 | 0 |
| 10871 | 2 | 0 | 0 | 0 | 0 | 1 (50) | 2 (100) | 0 | 1 (50) |
| 10876 | 3 | 0 | 0 | 0 | 1 (33) | 2 (67) | 2 (67) | 0 | 1 (67) |
| 10878 | 2 | 0 | 0 | 0 | 2 (100) | 2 (100) | 2 (100) | 0 | 2 (100) |
| 10883 | 2 | 0 | 0 | 0 | 0 | 1 (50) | 2 (100) | 0 | 0 |
| 10885 | 2 | 0 | 0 | 0 | 0 | 1 (50) | 2 (100) | 0 | 0 |
| 10886 | 3 | 0 | 0 | 0 | 0 | 0 | 0 | 0 | 0 |
| 10901 | 2 | 0 | 0 | 0 | 0 | 1 (50) | 1 (50) | 0 | 0 |
| 10902 | 2 | 0 | 0 | 0 | 0 | 0 | 2 (100) | 0 | 0 |
| 10915 | 2 | 0 | 0 | 0 | 0 | 1 (50) | 2 (100) | 0 | 0 |
| Other | 58 | 0 | 0 | 0 | 24 (41) | 26 (45) | 45 (78) | 1 (1.7)* | 23 (40) |

**Additional file 1: Table S1 *Neisseria gonorrhoeae* multiantigen sequence typing (NG-MAST) STs and their resistance to antimicrobials in Karachi, Pakistan, 2012-2014**

NG-MAST: *Neisseria gonorrhoeae* multiantigen sequence typing, ST: sequence type

*Azithromycin resistant isolate (MIC=4 mg/L) was assigned as ST858.
